# Fatal eosinophilic myocarditis and submassive hepatic necrosis in lamotrigine induced DRESS syndrome

**DOI:** 10.1186/s13223-023-00848-6

**Published:** 2023-10-25

**Authors:** Khanh Duy Doan, Adeyinka Akinsanya, Matthew Kuhar, Hector Mesa

**Affiliations:** 1 Department of Pathology and Laboratory Medicine, Temple University Hospital, Philadelphia, PA USA; 2 Department of Pathology and Laboratory Medicine, Indiana University School of Medicine, 350 W 11th St, Indianapolis, IN 46202 USA

**Keywords:** Lamotrigine, Drug hypersensitivity syndrome, Myocarditis, Massive hepatic necrosis, Thyroiditis, Autopsy

## Abstract

Drug Reaction with Eosinophilia and Systemic Symptoms (DRESS) syndrome is a rare but severe and sometimes fatal adverse drug reaction that is known to occur with a number of antiepileptic drugs. It often follows a prolonged clinical course, which can worsen even after discontinuing the causative drug and administering steroid treatment. Failure to promptly identify the delayed involvement of vital organs, such as the heart and liver, may result in irreversible organ failure and death. We report a case of a presumed sudden death of a young woman who had a documented history of a protracted intermittent hypersensitivity reaction to lamotrigine. Postmortem examination revealed the presence of eosinophilic myocarditis and submassive hepatic necrosis diagnostic of fatal DRESS syndrome that progressed despite early discontinuation of the medication and improvement of dermatologic and hematologic symptoms following steroid therapy.

## Background

Drug reaction with eosinophilia and systemic symptoms (DRESS) syndrome is a rare and potentially life-threatening adverse drug reaction. It manifests with cutaneous symptoms and internal organ involvement and is primarily associated with antiepileptic medications [1]. Although cardiac involvement occurs in up to 21% of DRESS syndrome cases [2], it is often overlooked due to its delayed onset following drug withdrawal and the dominance of cutaneous, hematologic, and liver dysfunction manifestations [3]. Symptomatic heart disease in affected patients commonly manifests with chest pain, tachycardia, dyspnea, and hypotension [4]. Diagnostic workup typically reveals EKG changes and elevated cardiac enzymes, though in some cases, non-specific gastrointestinal symptoms like nausea and vomiting may be the sole manifestations [4]. Myocarditis-associated mortality can reach 50%, with most patients succumbing within 60 days from symptom onset [5]. The most commonly used diagnostic criteria for DRESS syndrome from simple to complex include Bocquet’s criteria, the Japanese Consensus Group criteria, and the RegiSCAR scoring system, which have been summarized in Table 1 [6]. All systems require cutaneous rash, eosinophilia or reactive lymphocytosis, and internal organ involvement. The average time between starting or stopping a drug and development of DRESS syndrome is 2–6 weeks, but it may occur months after exposure [7]. Mild cases are managed by discontinuing the causative drug and providing supportive care, while severe cases may require high-dose systemic corticosteroids and other forms of immunosuppression, though the evidence supporting their use is not well-established [4–6] The low incidence/prevalence of DRESS syndrome, coupled with its variable clinical course and unpredictable delayed relapses, which usually occur during or after steroid taper, present significant challenges in determining the ideal monitoring, follow-up, and prevention of complications, as illustrated by this case.

**Table 1:** Diagnostic Criteria for DRESS

Criteria	Bocquet All criteria	Japanese Consensus Group All criteria	RegiSCAR Score < 2: negative; 2–3: possible; 4–5: probable; > 5: definitive
Cutaneous rash	Maculopapular	Rash > 3 wks. after exposure	Rash > 50% body surface (+ 1)
Eosinophilia or reactive lymphocytosis	Abs. Eos. > 1.5 K/µL	Abs. Eos. >1.5 K/µL; Atypical Lymphs. >5%	Abs. Eos 0.7 − 1.4 K/µL (+ 1) Abs. Eos. ≥1.5 K/µL (+ 2) Atypical Lymphs. (+ 1)
Organ involvement: Lymphadenopathy, hepatitis, nephritis, pneumonitis, carditis	Any	Any	(+ 1 for every organ)
Fever		≥ 38.5 ° C	Yes (0), No (-1)
Human herpesvirus type 6 (HHV-6) reactivation		Yes (typical) No (atypical)	
Negative work-up for autoimmune and infectious diseases			Yes (+ 1), No (0)
Biopsy compatible with DRESS			Yes (0), No (-1)
Resolution in ≥ 15 days			Yes (0), No (-1)

## Case presentation

A 39-year-old female presented to the emergency room with a generalized rash. She described that the rash started as a localized pruritic rash that spread slowly to cover much of her body, and that it began 5 days after the dose of lamotrigine, which she received for epilepsy, was increased from 75 mg to 100 mg. She immediately stopped taking the medication after the rash appeared. She was not taking any other medications and had no history of rheumatologic disease, recent travel, or recent vaccinations. Physical examination revealed a generalized erythematous maculopapular rash and lymphadenopathy. There were no areas of desquamation of the skin or mucosal involvement, and Nikolsky sign was negative, making Stevens-Jonhson syndrome, immunobullous disorders or infectious dermatoses unlikely. Laboratory workup showed elevated liver function tests: ALT 555 U/L (range 7–52), AST 269 U/L (range 13–39) and leukocytosis of 14.7 K/µL (range 3.6–10.6) with eosinophilia: 1.8 K/µL / 16% (range < 0.3 K/µL / < 6%). Rapid tests for Group A Streptococcus and Monospot test were negative. Serological testing for Epstein Barr virus, cytomegalovirus, hepatitis A, B and C were negative. Antinuclear antibodies (ANA) and antibodies against native double-stranded DNA (anti –dsDNA) were negative. Serum acetaminophen levels were within normal limits.

**Fig. 1:**
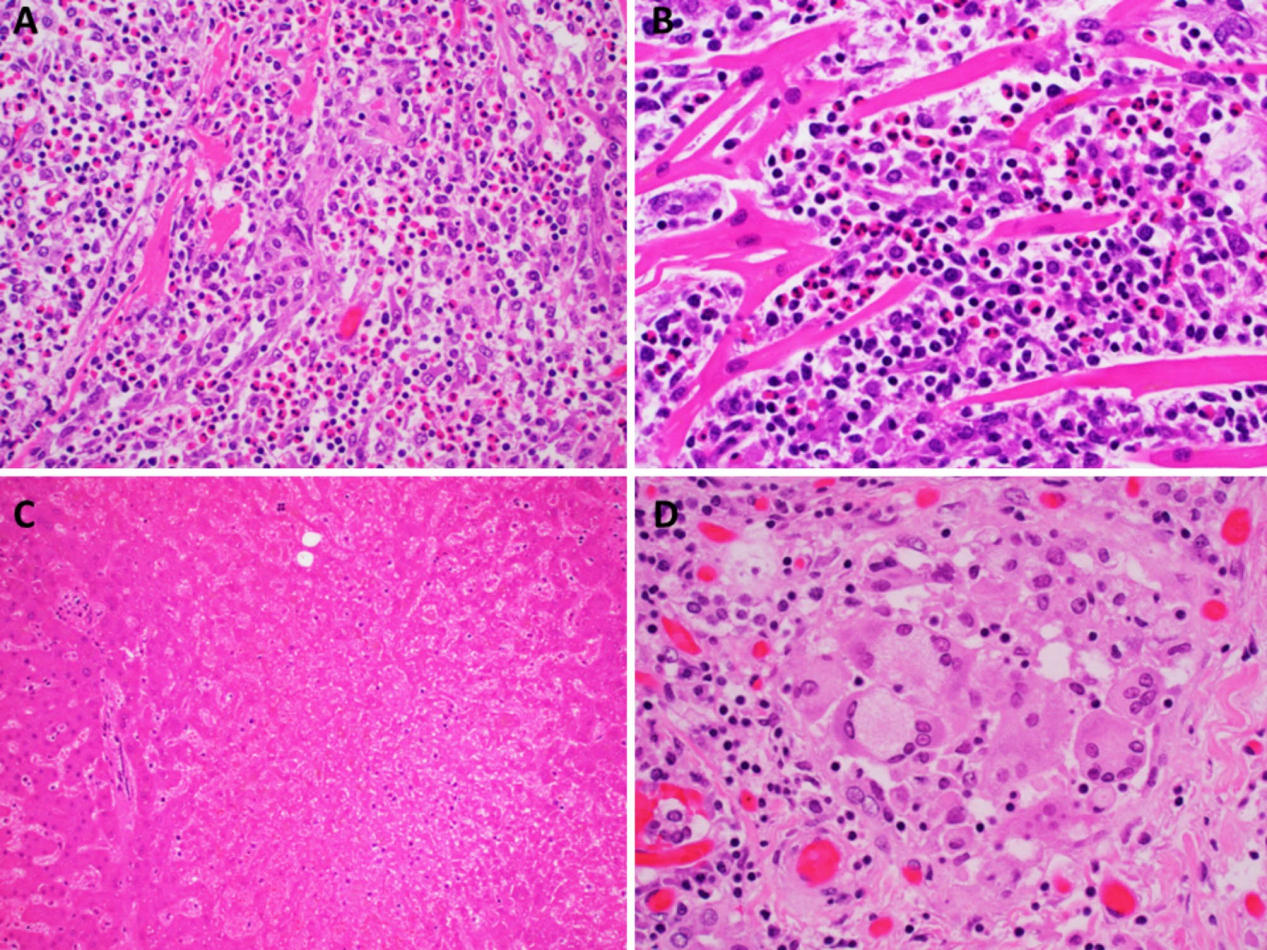
**A, B**. Myocardium. Intermediate and high-power sections from the heart showed an eosinophil-rich inflammatory infiltrate separating the myofibers with areas of complete loss of myocardial cells. (H&E, magnification 200X and 400X) **C**. Liver. The sections show extensive centrilobular coagulative necrosis. (H&E, magnification 100X) **D**. Thyroid. The thyroid follicles showed sloughing of follicular cells and infiltration by mononuclear cells with numerous multinucleated giant cells. (H&E, magnification 400X)

With a diagnosis of DRESS syndrome, the patient was discharged on a prednisone taper schedule starting with daily doses of 60 mg for 5 days, 40 mg for 3 days, 20 mg for 2 days, and 10 mg for 2 days. The rash improved initially, however, on day 7, while at 40 mg, the rash came back rapidly, involving much of her body, including palms and soles prompting a new visit to the emergency room. In addition to the rash she complained of malaise, chills, fever, nausea, vomiting, acute right upper quadrant abdominal pain, and shortness of breath, and was admitted. The physical examination showed normal vital signs and was unremarkable except for mild tenderness over the right-upper quadrant. Laboratory studies showed leukocytosis with neutrophilia: WBC: 15.3 K/uL, ANC: 9.9 K/uL (range 1.7–7.5) and prominent hyponatremia: 128 mmol/L (range 135–145), which was initially attributed to vomiting. The remaining blood tests were unremarkable. An abdominal ultrasound and magnetic resonance cholangiopancreatography performed to address the abdominal symptoms showed biliary tract dilation and mild bile duct wall enhancement suggesting possible acute cholangitis. The patient was managed with intravenous fluids, antiemetics, and antibiotics with no improvement in her clinical condition. On day 5 after admission, she slumped off the toilet while in the bathroom and went into cardiac arrest. Cardiopulmonary resuscitation was initiated. Possible ventricular fibrillation was identified by the automated external defibrillator and treated with cardioversion with brief non-sustained responses, and she expired. After authorization from the next of kin a postmortem examination was performed. The main autopsy findings were diffuse, severe necrotizing eosinophilic myocarditis, submassive centrilobular hepatic necrosis, and drug-induced thyroiditis (Fig. 1). There was indirect evidence of heart failure manifested by lung edema, effusions, and persistent electrolytic imbalance, and indirect evidence of impaired synthetic liver manifest by hypoalbuminemia and diffuse colonic mucosal hemorrhages.

## Discussion and conclusions

DRESS syndrome is most commonly caused by anticonvulsants and antibiotics [8]. The reported incidence of this syndrome in the general population ranges from 0.9/100,000 to 10/1,000,000 [9], and the incidence of Lamotrigine-associated DRESS syndrome ranges from 1 to 1,000 to 1 in 10,000 drug exposures and has been less frequently reported since the introduction of a gradual titration schedule in the 1990s [10]. In fact, our case stands out as the only fatal instance we encountered in our literature review since the year 2000. However, we found other cases with life-threatening complications, primarily severe liver toxicity, and less frequently eosinophilic myocarditis, affecting children and adults from both genders, which are summarized in Table 2 [11–14]. In these cases, severe organ complications emerged while steroids were being tapered, highlighting the importance of close monitoring during this phase of the treatment.

**Table 2:** Differential diagnosis of myocarditis associated with peripheral eosinophilia

Type	Diagnostic Clues
DRESS associated eosinophilic myocarditis	Temporal association with drug exposure, eosinophil-rich cutaneous manifestations
Eosinophilic granulomatosis with polyangiitis	Asthma, sinusitis, neuropathy, potential lung/renal syndrome, eosinophilic rich granulomas, positive p-ANCA test
Autoimmune myocarditis	Pre-existing autoimmune disorder (e.g., systemic lupus erythematosus), positive antinuclear antibodies (ANA) or extractable nuclear antigens (ENA) serologies, less conspicuous eosinophilia
Infectious myocarditis (parasites/protozoa/viruses)	History of recent viral infection or vaccination or specific exposures in endemic areas (e.g., travel to Latin America for Chagas disease), specific serological testing or viral/bacterial/parasitic PCR
Clonal hypereosinophilic syndromes	Persistent eosinophilia not responsive to steroids, evidence of organ involvement, bone marrow biopsy with pertinent molecular cytogenetic work-up: FIP1L1-PDGFRA or PCM1-JAK2 fusions, PDGFRA/B, FGFR1 rearrangements, KIT mutations

The underlying mechanism of DRESS syndrome is not fully understood. It is believed to involve an idiosyncratic drug-specific immune response, genetic deficiencies in detoxifying enzymes leading to the accumulation of drug metabolites, and reactivation of viral infections, including human herpesviruses 6 and 7, Epstein Barr virus, and cytomegalovirus [1, 5, 8]. DRESS is a delayed hypersensitivity reaction mediated by T helper 2 (TH2) cells associated with increased production of IL-4, IL-5, and IL-13, leading to recruitment and activation of eosinophils [8, 9].

Myocarditis associated with DRESS syndrome can present as either non-necrotizing eosinophilic myocarditis (EM) or acute necrotizing eosinophilic myocarditis (ANEM). Both types are characterized by the presence of an inflammatory infiltrate rich in eosinophils in the myocardium, with or without necrosis. The mortality rate for EM and ANEM is 40 and 50%, respectively. In EM, mortality is often attributed to cardiac arrhythmias or sudden death, while in ANEM, it is typically due to cardiogenic shock and refractory heart failure, as in the present case [2–5]. Differential diagnoses for drug-associated EM and ANEM include conditions such as eosinophilic granulomatosis with polyangiitis, autoimmune endomyocarditis, infectious myocarditis caused by parasites, protozoa, or viruses, as well as clonal hypereosinophilic syndromes (Table 3). DRESS-associated EM/ANEM is typically preceded by eosinophil-rich cutaneous manifestations and has a temporal association with specific drug exposures. Vasculitis and autoimmune myocarditis can be identified through serologic testing, such as Antineutrophil Cytoplasmic Antibodies (ANCA), ANA, anti-dsDNA, or when a preexisting diagnosis (e.g., Eosinophilic granulomatosis with polyangiitis or systemic lupus erythematosus) exists. Parasitic and protozoal infections are often endemic, and viral myocarditis is usually preceded by viral infections or vaccination. Evaluation for idiopathic hypereosinophilic syndrome should be done in patients with persistent, steroid-refractory peripheral eosinophilia, and it requires bone marrow examination with conventional and molecular cytogenetic work-up [1, 4, 6, 9, 15].

**Table 3:** Additional cases of lamotrigine induced severe DRESS syndrome

Ref.#	Age(yr.)/ Gender	Latency wks.	Presentation	Eosinophilia	Organ Involvement	Treatment	Outcome
11	39 / M	4	Fever, rash, facial edema	No	Liver, kidney, hematologic	Methylprednisolone, I.V. immunoglobulin	Recovered
12	21 / F	5	Fever, rash, lymphadenopathy	Yes	Liver, pancreas, lungs, hematologic	Methylprednisolone N-acetylcysteine	Liver transplant
13	12 / M	3	Fever, rash, facial edema, abdominal pain	Yes	Liver	Corticosteroids, antihistamines	Recovered
14	45 / F	12	Rash, chest pain, shortness of breath	No	Heart	Steroid, mycophenolate, mepolizumab, cyclosporine	Recovered

Around half of the patients with EM/ANEM present with symptoms such as dyspnea, fever, and heart failure, while less than 25% experience nausea and vomiting [4, 5]. In our case, the patient’s young age, stable vital signs upon admission, and prominent gastrointestinal symptoms during relapse led to an extensive evaluation for gastrointestinal and biliary tract disease, while the signs of heart and hepatic failure were overlooked.

Liver injury is a prevalent manifestation of DRESS, affecting up to 90% of cases [16]. The initial presentation of liver injury can vary, encompassing cholestatic type (37.1%), hepatocellular type (19.4%), and mixed type (27.4%). Among older individuals, the cholestatic type is more commonly observed and is often associated with anticonvulsants [17]. The understanding of the mechanisms underlying liver injury in DRESS is limited and is attributed to TH2-induced, and primarily IL-5-mediated recruitment of eosinophils, leading to eosinophil degranulation and the release of various inflammatory mediators and cytotoxic molecules. These include cationic proteins (major basic protein 1, eosinophil peroxidase, eosinophil cationic protein, eosinophil-derived neurotoxin), matrix metalloproteinases, proinflammatory cytokines, chemokines, and leukotrienes, resulting in toxic hepatitis. Similar mechanisms have been proposed for interstitial nephritis, pneumonitis, myositis, eosinophilic myocarditis, pancreatitis, thyroiditis, and encephalitis [16, 17].

At autopsy, there was also evidence of thyroiditis characterized by sloughing of follicular cells and infiltration of the follicles by mononuclear cells, including numerous multinucleated giant cells. In our review of the literature, thyroid dysfunction is a common side effect of classical antiepileptic drugs, but it appears to be rare with lamotrigine [18, 19]. We did not find any other reports of lamotrigine-induced thyroiditis.

This case highlights the insidious life-threatening complications of DRESS syndrome, which has earned it the nickname of “the great clinical mimicker” [20]. A definitive diagnosis of DRESS-associated EM/ANEM can be obtained through endomyocardial biopsy, and biopsy is recommended when there is suspicion or uncertainty regarding the diagnosis.

Due to the rarity of EM/ANEM no standardized treatment guidelines are available. The management typically involves discontinuation of the offending medication and a combination of immunosuppression, mechanical circulatory support, and heart failure medications, provided in an intensive care unit setting, tailored to the specific needs of individual patients [1–5]. Immunosuppressive therapy often includes high-dose steroids, while other immunosuppressive agents such as cyclosporine, mycophenolate, intravenous immunoglobulins, and rituximab have also been used [10–15, 21]. Considering the crucial role of eosinophils in organ toxicity, the utilization of IL-5 blockers like mepolizumab seems reasonable and is supported by anecdotal reports [14]. Antiviral medications are employed in patients with viral reactivations [22].

In conclusion, we have presented a case of a fatal acute necrotizing eosinophilic myocarditis caused by lamotrigine, which occurred during steroid taper several weeks after reactivation of a previously recognized DRESS syndrome. In this patient, myocarditis was missed due to the atypical clinical presentation and lack of systematic cardiac work-up.

The case highlights the challenges associated with DRESS syndrome, including its low incidence/prevalence, variable clinical course, and unpredictable severe relapses which usually occur during or after steroid taper. As illustrated by this report, judicious regular assessments of organ function, including cardiac, liver, kidney, and lung evaluations, are necessary, as are the development of standardized guidelines to optimize the monitoring, follow-up, and prevention of complications in these patients.

## Data Availability

Data sharing is not applicable to this article as no datasets were generated during the current study.
